# Effects of neuromuscular electrical stimulation during hemodialysis on muscle strength, functional capacity and postural balance in patients with end-stage renal disease: a randomized controlled trial

**DOI:** 10.1186/s12882-025-03994-8

**Published:** 2025-02-19

**Authors:** Amal Machfer, Nadia Fekih, Achraf Ammar, Hayfa Ben Haj Hassen, Wael Daab, Hassen Ibn Hadj Amor, Mohamed Amine Bouzid, Hamdi Chtourou

**Affiliations:** 1 Research Laboratory: Education, High Institute of Sport and Physical Education, University of Sfax, Sport et Santé, EM2S, LR19JS01, Motricité, Tunisia; 2 Department of Training and Movement Science, Institute of Sport Science, Johannes Gutenberg-University Mainz, 55099 Mainz, Germany; 3 Research Laboratory, Molecular Bases of Human Pathology, Faculty of Medicine of Sfax, University of Sfax, Sfax, LR19ES13, 3029 Tunisia; 4 Interdisciplinary Laboratory in Neurosciences, Physiology and Psychology: Physical Activity, Health and Learning (LINP2), Faculty of Sport Sciences), UFR STAPS, Paris Nanterre University, Nanterre, 92000 France; 5 High Institute of Sport and Physical Education of Sfax, University of Sfax, Sfax, Tunisia; 6 College of Sport Science, University of Kalba, Sharjah, United Arab Emirates; 7 Department of Cardiology, Tahar Sfar Hospital, Mahdia, Tunisia; 8 Activité Physique, Sport et Santé, UR18JS01, Observatoire National du Sport, Tunis, 1003 Tunisia

**Keywords:** Chronic kidney disease, Electric stimulation therapy, Hemodialysis

## Abstract

**Background:**

Hemodialysis patients (HD) have a limited physical capacity and this often means low adherence to rehabilitation programs based on conventional exercise. This study investigated the effectiveness of neuromuscular electrical stimulation (NMES) during HD therapy on muscle strength, functional capacity and postural balance in HD patients.

**Methods:**

Twenty-two HD patients were randomly assigned to a control group (CG) or a neuromuscular electrical stimulation training group (NSTG). The NSTG underwent NMES on the quadriceps muscle during HD sessions for 12 weeks, three times per week (40 min per session. Center of pressure (COP) displacement in the mediolateral direction (COPx), in the anteroposterior direction (COPy), and the COP area (COP area) were recorded using a stabilometric platform. Timed Up and Go test (TUG) and Sit to Stand (STS30) tests, 6-minute walking test (6MWT), and the maximal voluntary contraction (MVC) were measured before and after the intervention in both groups.

**Results:**

There was a significant increase in MVC (+ 24.5%; *P* < 0.01), 6MWT (+ 9.8%; *P* < 0.05) and STS30 (+ 25.6%; *P* < 0.01) performance in the NSTG following the NMES intervention period. A significant reduction was observed in TUG (-11.8%; *P* < 0.01), COPx(-20.1%; *P* < 0.05) and COPy (-24.7%; *P* < 0.01) following the intervention period only in the NSTG. However, no significant changes were observed in the CG following the intervention period.

**Conclusion:**

This study supports the effectiveness of intradialytic NMES to improve muscular strength, functional capacity and postural balance in HD patients. Given the limited implementation of exercise programs in dialysis clinical practice, NMES during HD sessions offers a novel therapeutic alternative to enhance physical condition and quality of life in these patients.

**Trial registration:**

Pan African Clinical Trial Registry Identifer: PACTR202206634181851 Registered on 21/06/2022. Registered trial name: Beneficial Effect of Intradialytic Electrical Muscle Stimulation in Hemodialysis Patients.

**Supplementary Information:**

The online version contains supplementary material available at 10.1186/s12882-025-03994-8.

## Background

Chronic kidney disease (CKD) is characterized by a persistent functional impairment of the kidneys lasting for at least three months, with significant health implications [1]. The kidneys play a vital role in maintaining homeostasis by regulating electrolyte and water balance, which directly impact the functioning of vital organs. As a result, a decline in kidney function is often linked to various metabolic disturbances, particularly in the most advanced stage when patients undergo regular hemodialysis therapy (HD) [2].

HD therapy mandates patients to attend sessions at least three times a week, fostering a sedentary lifestyle and frailty, which often leads to HD-related muscle weakness. Moreover, it has been reported in the literature that patients undergoing HD therapy (HD patients) have significantly reduced physical function and activity levels due to a number of comorbidities which are mechanistically linked including cardiovascular disease, bone-mineral abnormalities, and muscle catabolism [3]. Given these comorbidities, it is not surprising that HD patients have impaired mobility and balance [4], which is linked to elevated fall risk [5].

Exercise interventions should be promoted in this population to mitigate or reverse the adverse consequences of the disease, ultimately enhancing patients’ quality of life [6]. Although exercise can be beneficial, participation in physical activity programs among HD patients has been limited. The main barriers are limited time, post-HD fatigue, and difficulty accessing exercise programs, which are often offered in rehabilitation or cardiovascular centers rather than nephrology departments or dialysis clinics [7]. Additionally, HD often leaves patients too fatigued to engage in physical activity, making interventions outside the HD process difficult. As a result, an alternative intervention is needed to improve mobility and promote physical activity in HD patients.

Due to the high prevalence of hypertension, diabetes, and cardiovascular mortality, the optimal way to decrease morbidity and mortality is to increase physical activity. Conventional training methods based on systemic workload pose increased hemodynamic risks, such as elevated blood pressure, heart rate fluctuations, and acute cardiovascular complications, including arrhythmiasfor HD patients [8]. Thus, safer alternatives to current rehabilitation procedures are necessary. Neuromuscular electrical stimulation (NMES) serves as a promising alternative for this population, offering reduced hemodynamic risks compared to conventional training and demonstrating a low risk of adverse events [9].

NMES is a technique that consists of low-intensity electrical stimulation of skeletal groups with electrodes placed on the skin. These impulses stimulate the nerves to send signals to a specifically targeted muscle, which reacts by contracting, as it would with normal muscular activity [10]. It is widely used in healthy individuals who participate in physical activities or sport to improve physical condition and muscular strength.

Considering the peripheral muscle dysfunction in HD patients, which affects both postural balance and exercise capacity [11, 12], the effects of training program with NMES during HD therapy (intradialytic) in HD patients should be further investigated. Studies of NMES in HD patients available in the scientific literature usually used very low or very high frequency NMES therapeutic strategies and the reported results about its effects on muscle strength and functional capacity are diverging. A randomized study conducted by Dobsak et al. [8] showed a positive effects of intradialytic electrical stimulation on quadriceps and calf with a frequency of 10 Hz on peripheral muscle strength and exercise capacity (6 min walking test) in HD patients. However, Schardong et al. [9]reported no effects on 6 min walking test in HD patients following 8 weeks of intradialytic NMES intervention with a frequency of 80 Hz. Moreover, to our knowledge, no data are available about intradialytic NMES effects on postural balance in HD patients. Therefore, this study aimed to evaluate the effects intradialytic NMES intervention on peripheral muscle strength, exercise capacity, as well as postural balance in HD patients.

## Methods

### Participants

**Fig. 1:**
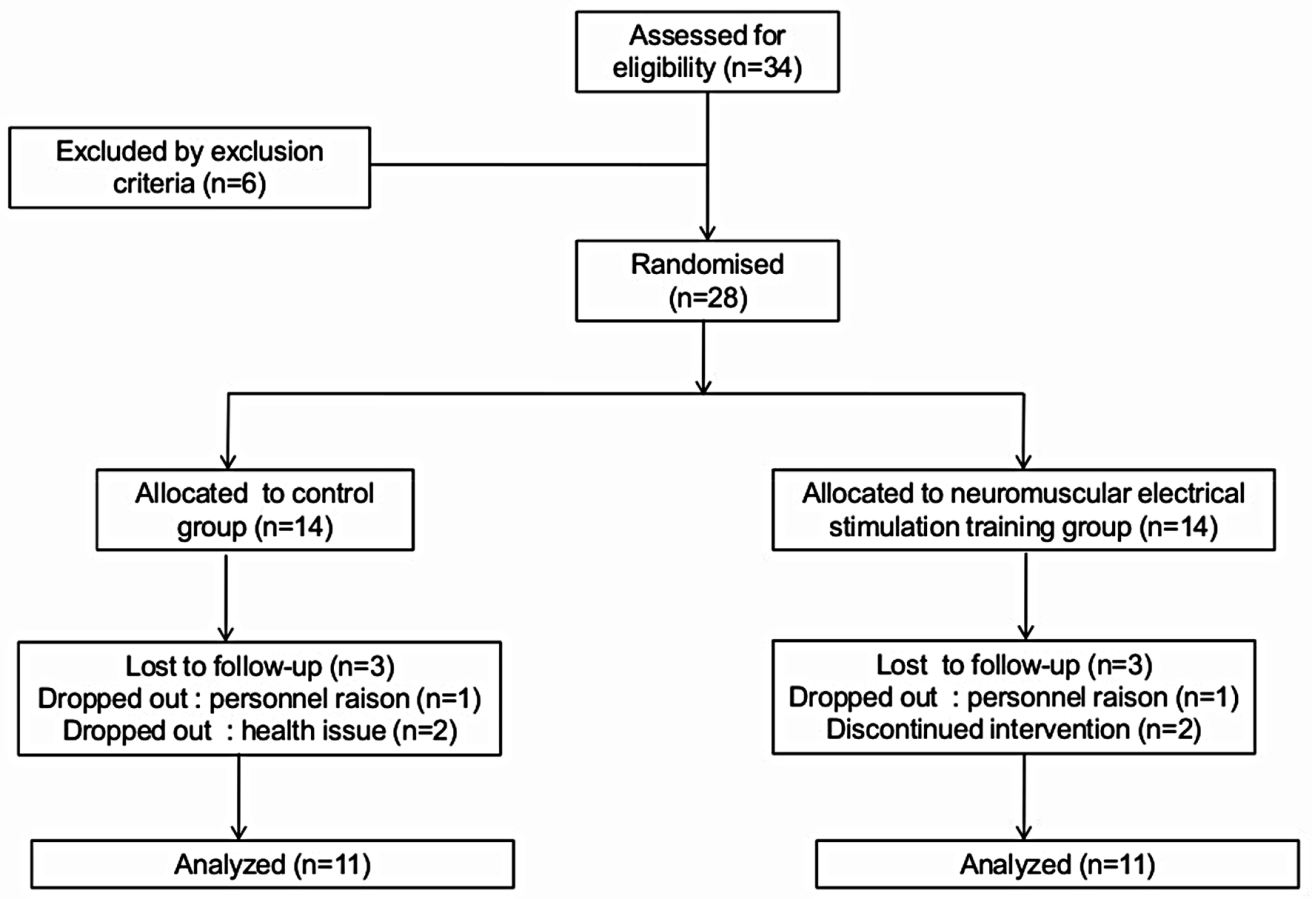
Flowchart of study participants

All subjects gave informed consent for study participation. The study received approval from the Regional Research Ethics Committee (CPP SUD N° 11/2019) and registered with the Pan African Clinical Trial Registry (PACTR202206634181851) and followed the ethical principles of the Declaration of Helsinki. A total of 34 HD patients were eligible for the study based on data from their medical records. Entry criteria included receipt of chronic dialysis therapy for 12 months or longer. Six were excluded from the study due to the exclusion criteria [i. e., active coronary artery disease (*n* = 1), > 3 L of fluid accumulation between hemodialysis (*n* = 1), intradialytic blood pressure of 180 mmHg systolic or 95 mmHg diastolic (*n* = 1), hemoglobin < 9.0 g/dL (*n* = 2) and ischemic cardiac event (*n* = 1)]. Study procedures were completed on 28 participants and 6 of them were excluded because of personnel raison (2) and health issue (2). One patient in the NSTG group.

reported experiencing cramps three times, which caused uncertainty and led to them dropping out of the study. Another patient experienced hip pain unrelated to NMES but decided to discontinue the study. Thus 22 HD patients were included in the data analysis (Fig. 1).

### Study design

This study consists of a randomized clinical trial conducted in HD patients that were submitted to NMES. Eligible patients were randomized into two groups: control group (CG) (*n* = 11) and neuromuscular electrical stimulation training group (NSTG) (*n* = 11) (Table 1). Randomization occurred through data generated by random.org online software (www.random.org). The sequence of numbers was generated by researchers “blind” to the study after the selection of patients for eligibility criteria and disclosed prior to the start of the intervention program. Further details are provided in online supplemental methods.

**Table 1:** Descriptive data of the participants (mean ± SD)

	CG group (*n* = 11)	NSTG group (*n* = 11)	*p*
**Anthropometric date**			
Age (years)	35.1 ± 5.3	38.5 ± 4.8	0.53
Weight (Kg)	69.3 ± 6.2	70.6 ± 2.9	0.81
Height (m)	1.7 ± 0.3	1.7 ± 0.1	0.99
BMI (%)	23.2 ± 2.5	23.9 ± 1.8	0.88
**Physical activity score**	3.6 ± 1.9	4.1 ± 1.8	0.61
Comorbidities			
Diabetes mellitus type 2 (%)	1 (9%)	2 (18%)	-
Hypertension (%)	2 (18%)	2 (18%)	-
**Clinical parameters**			
Time on dialysis (months)	36.1 ± 11.6	34.2 ± 10.3	0.54
eGFR (ml/min/1.73 m^2^)	8.1. ± 2.5	8.4. ± 1.9	0.44
Hb (mg/dl)	108.1 ± 13.1	108.1 ± 13.1	0.85
Blood pressure (systolic) (mmHg)	137.2 ± 12.2	143.35 ± 15.6	0.39
Blood pressure (diasystolic) (mmHg)	80.4 ± 5.5	82.5 ± 9.1	0.28
Heart rate at rest (bpm)	68.8 ± 9.1	72.3 ± 8.3	0.44
Kt/V	1.37 ± 0.04	1.43 ± 0.11	0.51
Charlson Comorbidity Index (score)	2.09	2.18	0.76

BMI body mass index, eGFR estimated glomerular filtration rate, Hb hemoglobin, Kt/V dialysis efficiency

The baseline and final assessments were enrolled on the non-hemodialysis days. The final assessment was performed after 12 weeks of the training protocol. Subjects reported to the laboratory on two occasions, once for a familiarization session and once for the experimental session, during which peripheral muscle strength, postural balance and functional capacity were assessed. All these outcomes measurements were evaluated at baseline and after the intervention period (12 weeks) for all participants on the non-dialysis days by the same-trained professional experimenter who was blinded to the participant’s group.

### Admission visit

4 days before the experiment, subjects attended a familiarization session, during which they were introduced to the experimental procedures. On arrival at the laboratory, anthropometric variables were measured and each subject was instructed on achieving maximal strength levels using an isometric dynamometer. Participants were also assessed for physical activity levels, leg dominance and Charlson Comorbidity Index (CCI) [13].

### Intervention protocol

All patients underwent the standard HD care, but patients in the NSTG group additionally received an intradialytic NMES of the quadriceps muscles of both lower extremities. Each participant of NSTG completed at 36 NMES training sessions at the rate of 3 sessions per week. Each NMES sessions lasted 40 min and comprised 80 isometric contractions for each knee extensor muscles. Each contraction lasted 10 s and was followed by a 20 s resting period. The participant’s trunk was set at a 120 ° angle and leg flexion at 60 °, which corresponds to the position where maximal force can be obtained (0 ° corresponding to complete leg extension) [14].

The contractions were produced with a portable programmable electric stimulator (Genesy 1200 PRO, Globus Italia, Codognè, Italy), which delivered 400 µs rectangular and biphasic-wave pulsed currents at a frequency of 50 Hz. We chose rectangular waves associated with long pulse durations (300–400 µs) because they appear to produce the most powerful contraction of the quadriceps muscle group [15]. The 50 Hz stimulation frequency falls within the 50–120 Hz range shown to be the most efficient for strength training [16]. NMES was produced using self adhesive bipolar electrodes (4 electrodes for each leg, model MyoTrode, 5 × 5 cm; GLOBUS Italia SRL). Maximum intensity was achieved by encouraging the patient to bear with the maximum painless level of stimulation, thus reaching a tolerable and effective muscle contraction. Further details are provided in online supplemental methods.

2 negative electrodes were placed close to the proximal insertion of the vastus lateral (VL) and the vastus medialis (VM) muscles, over the femoral triangle of each leg, 1–3 cm below the inguinal ligament. Pairs of positive electrodes were placed as close as possible to the motor points of the VL and VM muscles. The motor points were determined by moving a probe over the skin surface to find the lowest threshold for stimulation [17]. Electrodes were not removed or replaced during the session. All patients were asked to continue their lifestyle as usual. The training procedures were realized by the trained study assistants and supervised by the medical staff.

### Study outcomes

The participants performed three maximal voluntary contraction ofthe knee extensors.

each lasting 5 s with a 3 min rest period between the attempts. They were seated on an isometric dynamometer (Good Strength, Metitur, Finland) whose reliability and validity were documented [18]. Participants were seated with a 90° knee fexion angle from full extension with a cuff attached to a strain gauge of the dynamometer and were stabilized with safety belts strapped across the chest, thighs, and hips, to avoid lateral, vertical, or frontal displacements. This cuff was adjusted 2 cm above the lateral malleolus using a noncompliant Velcro strap for recording of quadriceps force. All measurements were taken from the participant’s dominant leg.

### Assessments of postural balance

Participant’s standing postural balance was assessed using a static stabilometric platform (PostureWin©, TechnoConcept^®^, Cereste, France; 14 Hz frequency, 12-bits A/D conversion) which recorded the displacements of the center of pressure (COP) and whose reliability and validity were documented [19]. Participants were instructed to stand erect, as motionless as possible, on a normal comfortable posture, with eyes open looking straight ahead at a cross marked at approximately eye level 3 m away and barefoot with feet shoulder width apart on the platform with the arms by their sides and head right. Each participant was requested to keep a quiet stance during 25.6 s following the French Posturology Association norms [20]. To evaluate postural balance of our participants, three COP sways parameters were analyzed in this study: The COP area, the COP lengths corresponding to the sum of COP displacement in the medio-lateral (COPx) and in antero-posterior (COPy).

### Assessments of functional capacity

#### Time Up and go test (TUGT)

Functional mobility was assessed using the Timed Up and Go test (TUGT). Reliability and validity were previously demonstrated in chronic kidney diseases patients [21]. Participants were timed as they rose from a 45 cm-high straight-backed chair, walked 3 m, turned, and returned to their original sitting position [22]. The time (s) to accomplish the TUGT was calculated for each participant.

#### Sit to stand (STS30)

Lower-body strength and endurance were determined using the 30-second Sit To Stand test (STS30) whose reliability and validity were documented in HD patients [23]. Participants were asked to sit in a standard height chair with their arms crossed over the chest, then stand fully and sit down again as many times as possible within 30 s [24].

#### Six-minute walk test (6-MWT)

The Six-minute walk test (6-MWT) was performed following the recommendations of the American Thoracic Society [25]. Reliability and validity of the 6-MWT in HD patents has been previously demonstrated [26]. During the test, participants were instructed to walk as fast as possible during 6 min on a flat of 30-m long track. They were allowed to stop and to have a rest during the test, but were instructed to resume walking as soon as they felt able to do so.When the test was completed, the total distance travelled was registered.

### Statistical analyses

The sample size calculation was based on a previous investigation documenting NMES training effects in HD patients compared to control group [8]. Assuming an effect size of 0.93, *α* = 0.05, and *β* = 0.8, the minimum number of participants required to establish a significant difference in maximal voluntary force between before and after NMES intervention and between the two groups using two-way repeated-measures ANOVA, was calculated at 10 per group (G*power, version 3.1.9.4).

Statistical analyses were performed using Statistica for Windows software (version 12.0). The normality of every dependent variable and homogeneity of distribution variances (equal variance) was confirmed using Shapiro-wilk test and the Levene test, respectively. Participant characteristics were compared using independent t-tests. Two-way ANOVA (group x training) was used was used to analyze data. To assess the ANOVA practical significance, partial etasquared (ηp2) was calculated. When a significant difference was found, multiple-comparison analysis was performed with the Bonferroni post hoc test. Results are reported as the mean ± SD and statistical significance was set at *P* < 0.05.

## Results

### Muscle strength

Concerning MVC, statistical analysis demonstrated a significant interaction effect (group*training) (F_[1,10]_ = 22.7, *P* < 0.01, ηp^2^ = 0.71). MVC at baseline was similar between CG (379.90 ± 31.51 N) and NSTG (401.27 ± 24.90 N) (*P* = 0.59). Post hoc analysis demonstrated that MVC values significantly increased following the NMES training period in NSTG (401.27 ± 24.90 N to 495.36 ± 20.33 N; +24.5%) (*P* < 0.01). However, no significant changes were observed in MVC values following the NMES training period in CG (379.90 ± 31.51 N to 398.30 ± 19.1 N; +5.5%) (*P* = 0.23). (Table 2).

**Table 2:** Summary for muscle strength (maximal voluntary contraction: MVC), six-minute walk test (6-MWT), Time Up and go test (TUGT) and sit to stand test (STS30) data in control (CG) and neuromuscular electrical stimulation training (NSTG) groups before and after the intervention period (Mean ± SD)

	CG		NSTG		Effects	ηp2
	Pre training	Post training	Pre training	Post training		
MVC (N)	379.9 ± 31.5	398.3 ± 19.1	401.2 ± 24.9	495.3 ± 20.3^#^*	Interaction *P* < 0.01 Training *P* < 0.01 Group *P* < 0.01	*0.71*
						*0.67* *0.52*
6-MWT (m)	513. 2 ± 79.2	500.2 ± 55.7	510.3 ± 56.4	548.9 ± 53.3^#^*	Interaction *P* < 0.01 Training *P =* 0.02 Group *P* < 0.01	*0.64*
						*0.33* *0.42*
TUGT (s)	6.03 ± 0.67	6.06 ± 0.85	6.2 ± 0.95	5.5 ± 0.48#*	Interaction *P* < 0.01 Training *P* < 0.01 Group *P =* 0.04	*0.60* *0.24*
						*0.35*
STS30 (A.U)	12.2 ± 1.7	13.1 ± 1.2	12.6 ± 1.3	15.8 ± 1.8#*	Interaction *P* = 0.02 Training *P* < 0.01 Group *P* = 0.03	*0.42* *0.82*
						*0.63*

∗*p* < 0.05 vs. baseline, # *p* < 0.05 vs. control group

### Postural balance

As shown in Table 3, statistical analysis showed no significant interaction effect (group*training) for COP area (F_[1,10]_ = 2.01, *P* = 0.21, ηp^2^ = 0.28). In addition, we noted only a significant training effect for COPx (F_[1,10]_ = 10.53, *P* = 0.02, ηp^2^ = 0.67) and COPy (F_[1,10]_ 8.50, *P* < 0.01, ηp2 = 0.35). Post hoc analysis revealed a significant reduction in COPx (-20.1%) and COPy (-24.7%) values following the training period in the NSTG (*p* < 0.05). However, no significant changes were observed in COPx (-7.2%) and COPy (-7.1%) for CG participants.

**Table 3:** Summary for postural balance data data in control (CG) and neuromuscular electrical stimulation training (NSTG) groups before and after the intervention period (Mean ± SD)

	CG		NSTG		Effects	ηp2
	Pre training	Post training	Pre training	Post training		
COP area (mm^2^)	154.67 ± 41.00	163.33 ± 35.49	198.11 ± 41.47	139.89 ± 36.66	Interaction *P* = 0.21 Training *P =* 0.26 Group *P =* 0.58	*0.28*
						*0.24* *0.06*
COPx (mm)	179.83 ± 25.96	166.50 ± 21.45	178.89 ± 30.30	142.56 ± 40.31*	Interaction *P* = 0.49 Training *P =* 0.02 Group *P =* 0.82	*0.09*
						*0.67* *0.01*
COPy (mm)	255.33 ± 26.21	237.67 ± 30.51	284.67 ± 36.68	214.78 ± 40.54*	Interaction *P* = 0.30 Training *P* < 0.001 Group *P =* 0.45	*0.20* *0.78*
						*0.11*

COP: center of pressure, COPx: COP displacement in the medio-lateral plan, COPy: COP displacement in the antero-posterior plan

∗*p* < 0.05 vs. baseline, # *p* < 0.05 vs. control group

### Functional capacity

Statistical analysis demonstrated a significant interaction effect (group*training) (F_[1,10]_ = 18.45, *P* < 0.01, ηp^2^ = 0.64) for the 6-MWT test. Post hoc analysis showed that performance in 6-MWT significantly increased following the NMES intervention period in NSTG (510.87 ± 60.15 m to 548.35 ± 53.99 m; +9.8%) (*P* = 0.02). However, no significant changes were observed in 6-MWT values in CG (513.25 ± 79.05 m to 500.15 ± 55.42 m; -2.2%) (*P* = 0.11) (Table 2).

Regarding TUGT, our results showed a significant interaction effect (group*training) (F_[1,10]_ = 15.22, *P* < 0.01, ηp^2^ = 0.60). Post hoc analysis showed no significant changes in TUGT performance following the intervention period in the CG (6.03 ± 0.67s to 6.06 ± 0.35s;- 0.66%) (*P* = 0.82). However, we noted a significant reduction in TUGT in the NSTG following the intervention period (6.24 ± 0.95s to 5.50 ± 0.48 s;-11.8%) (*P* < 0.01) (Table 2).

Finally, we noted a significant interaction effect (group*training) (F_[1,10]_ = 7.29, *P* = 0.02, ηp2 = 0.42) for the STS30 test. Post hoc analysis revealed a significant increase in STS30 test performance in the NSTG following the intervention period (12.64 ± 1.36 to 15.89 ± 1.81; +25.6%) (*P* < 0.01). Likewise, no significant changes were observed in the STS30 test for the CG (12.29 ± 1.72 to 13.14 ± 1.22; +7.8%) (*P* = 0.59) (Table 2).

## Discussion

Reduced exercise capacity is a prominent feature in HD patients. The ability to attenuate functional decline and postural imbalance is crucial to improving quality of life and morbidity in this population. Rehabilitative strategies are poorly defined in HD patients despite promising results with intradialytic exercise. A particular challenge is the provision of exercise therapy for HD patients who are unable to perform conventional dynamic training. Here we investigated the effectiveness of NMES training during HD on muscle strength, functional capacity and postural balance in HD patients. Our data show that, compared to CG, NMES promotes increased in lower limbs strength and improved functional capacity and postural balance in NSTG group.

Our results showed a significant increase in muscle strength of the lower limb assessed by dynamometry in NSTG group following the NMES intervention period. Our findings align with those of Dobsak et al. [8]who reported significant gains in maximum leg strength in 32 HD patients after three weekly sessions over 20 weeks, with each electrical stimulation session lasting 60 min. In the same way, Schardong et al. [9] showed an increase in lower limb strength in HD patients. Those authors used a shorter treatment time and shorter electrical stimulation session time (8 weeks; 20–34 min/session) using the progression of overload through reduction of the rest time and increase of stimulation time over the weeks. The precise mechanisms underlying the observed improvements cannot be determined from the current data. However, it’s possible that improvement in muscle strength may have been achieved through increase in muscle bulk as a result of repetitive contractions. It may also have arisen from facilitation of spinal motoneuron pools via stimulation of afferent pathways, increased sensitivity of neural synapses, and better synchronization of motor unit firing patterns [27].In addition, the selective recruitment of large fast-twitch type II fibers over the slow-twitch type I fibers with NMES could also be implicated in the improvement in muscle strength observed in NSTG [28].

Functional tests such as the 6MWT, STS30, and TUGT are widely used in clinical practice to assess functional capacity, though significant variability in test outcomes among HD patients is well documented [8, 9, 29]. In our study, the NMES intervention produced a significant increase in the distance walked in the 6MWT (+ 9.8%), STS30 (25.6%) and TUGT (11.8%) only in the NSTG group. This improvement highlights the muscular activation of the quadriceps muscles and the effectiveness of NMES in strengthening lower extremities.

These findings fully agree with the results of recent studies, which reported significant increase in walked distance and improvement of functional parameters after NMES in HD patients.Simo et al. [23] reported an improvement in functional capacity and quality of life in a group of 11 HD patients after 12-weeksof NMES of both quadriceps muscles during HD sessions. Identical results were obtained in a study previously published by Suzuki et al.regarding the role of NMES on improving functional capacity in HD patients [30]. Therefore, ourstudy brings further favorable data regarding the safety, efficacy and tolerability of intradialytic NMES in HD patients.

Moreover, we found that NMES improved static postural balance outcomes (COPx and COPy) in the NSTG group. To our knowledge, our study is the first to investigate the effects NMES during HD on muscle postural balance in HD patients. Our result agrees well with previous findings obtained in elderly patients by Amiridis et al. [25]and Mignardot et al. [26] who used a force plate to measure COP variations while standing and found that medio/lateral COP displacement improved 50.0% after NMES intervention period [31, 32]. Dos Santos et al. [33]. showed in a review that NMES training improved muscle balance (quadriceps and hips) in people with patellofemoral dysfunction. In the same way, inpatients with chronic obstructive pulmonary disease, Mekki et al. [34] found an improvement in postural outcomes after NMES intervention period.In the present study, improved static postural balance with NMES could be explained by an increase of muscle strength [31]. In fact, it is well documented that poor balance in HD patientsis associated with muscle weakness [35]. Therefore, improvement of muscle strength would have a positive impact on restoring balance deficits. Moreover, NMES could improve postural balance (COPy and COPx) by enhancing of the somatosensory function of the lower limbs. In fact, it has been reported that NMES could enhance the patients’ ability to integrate the somatosensory and vestibular inputs, becoming less reliant on the visual input while applying appropriate sensory strategies to control their posture and prevent falls [36].

### Methodological limitations

Some limitations are inherent to the experimental protocol of this study warrant mention. First, the number of participants was low due to the difficulty of the recruitment of HD patients. The study protocol involved several tests, which many HD patients were unwilling to undergo. Second, the control group received standard HD care without intervention. While this is a common approach, the absence of a sham NMES protocol or alternative intervention limits the ability to isolate NMES-specific effects from placebo or motivational influences. Third, participants in the present study were younger (mean age: 36 years) compared to HD subjects in previous studies (commonly aged 50–70 years in dialysis units), which may limit the generalizability of these findings.

## Conclusion

In summary, NMES can be an initial strategy of rehabilitation and treatment for this population, as these patients have low tolerance to exercise overload often rendering conventional training unfeasible. For the many instances in which conventional dynamic exercise is prevented by comorbidity and fatigue, NMES is a promising alternative strategy to improve quality of life and physical status in HD patients. This exercise modality can be easily administered on dialysis units. Further studies should focus on observing the effect of different types of NMES programs (with various frequency and intensity) on physical capacity in HD patients in order to identify the optimal NMES training protocol for this population.

## Electronic supplementary material

Below is the link to the electronic supplementary material.

Supplementary Material 1

## Data Availability

The data may be shared upon reasonable request to the corresponding author if the request is accepted by the Regional Research Committee for Medical and Health Research Ethics and the local Data Protection Official.

## Abbreviations

NMES: Neuromuscular electrical stimulation
HD: Hemodialysis
COP: Center of pressure
TUG: Timed Up and Go test
STS30: Sit to Stand test
6MWT: 6-minute walking test
MVC: Maximal voluntary contraction
CKD: Chronic kidney disease
