# Supplementary material for: Effects of neuromuscular electrical stimulation during hemodialysis on muscle strength, functional capacity and postural balance in patients with end-stage renal disease: a randomized controlled trial
Source: BMC Nephrol. 2025 Feb 19;26:86. doi: 10.1186/s12882-025-03994-8 (PMC11837610; doi:10.1186/s12882-025-03994-8)
Supplement: Supplementary file 1 — Supplementary Material 1 [file 12882_2025_3994_MOESM1_ESM.docx]

Supplemental Methods: Additional Methodology Details

**Participants**

All subjects gave informed consent for study participation. The study received approval from the Regional Research Ethics Committee (CPP SUD N° 11/2019) and registered with the Pan African Clinical Trial Registry (PACTR202206634181851) and followed the ethical principles of the Declaration of Helsinki. A total of 34 HD patients were eligible for the study based on data from their medical records. Entry criteria included receipt of chronic dialysis therapy for 12 months or longer. Six patients were excluded from the study due to the exclusion criteria [i. e., active coronary artery disease (n = 1), > 3 L of fluid accumulation between hemodialysis (n = 1), intradialytic blood pressure of 180 mmHg systolic or 95 mmHg diastolic (n = 1), hemoglobin < 9.0 g/dL (n = 2) and ischemic cardiac event (n = 1)]. Study procedures were completed on 28 participants and 6 of them were excluded because of concerns with exercise testing. Thus 22 ESRD patients were included in the data analysis (Figure 1).

**Study design**

Eligible patients were randomized into two groups: control group (CG) (n= 11) and neuromuscular electrical stimulation training group (NSTG) (n=11) (Table 1). Randomization was generated by random.org online software. The baseline and final assessments were enrolled on the non-hemodialysis days. The final assessment was performed after 12 weeks of the training protocol. Subjects reported to the laboratory on two occasions, once for a familiarization session and once for the experimental session, during which peripheral muscle strength, postural balance and functional capacity were assessed.

Randomization
Eligible patients were randomized into two groups: control group (CG) and neuromuscular electrical stimulation group (NMESG). Randomization occurred through data generated by random.org online software. The sequence of numbers was generated by researchers “blind” to the study after the selection of patients for eligibility criteria and disclosed prior to the start of the intervention program.

**Admission visit**

4 days before the experiment, subjects attended a familiarization session, during which they were introduced to the experimental procedures. On arrival at the laboratory, anthropometric variables were measured and each subject learned to achieve maximal strength level using an isometric dynamometer. Participants were also assessed for physical activity levels, leg dominance and Charlson Comorbidity Index (CCI) (Charlson, Pompei et al. 1987).

Physical activity score

Physical activity history was assessed using the physical activity questionnaire of Baecke et al. (1982). Participants of both groups were interviewed about the frequency of the physical activities they performed during the last 12 months. This test comprises 16 questions, including 3 habitual Physical Activity scores from the past 12 months: 1) occupational physical activities score (8 questions); 2) physical exercises in leisure (PEL) score (4 questions); 3) leisure and locomotion physical activities (LLA) score (4 questions). All responses were precoded on 5-point scales

**Intervention protocol**

All patients underwent the standard HD care, but patients in the NSTG group additionally received an intradialytic NMES of the quadriceps muscles of both lower extremities. Each participant of NSTG completed at 36 NMES training sessions at the rate of 3 sessions per week. Each NMES sessions lasted 40 min and comprised 80 isometric contractions for each knee extensor muscles. Each contraction lasted 10 s and was followed by a 20 s resting period. The participant’s trunk was set at a 120 ° angle and leg ﬂexion at 60 °, which corresponds to the position where maximal force can be obtained (0 ° corresponding to complete leg extension) (Maffiuletti, Cometti et al. 2000).

The contractions were produced with a portable programmable electric stimulator (Genesy 1200 PRO, Globus Italia, Codognè, Italy), which delivered 400 µs rectangular and biphasic-wave pulsed currents at a frequency of 50Hz. We chose rectangular waves associated with long pulse durations (300–400 µs) because they appear to produce the most powerful contraction of the quadriceps muscle group (Bowman and Baker 1985). The 50Hz stimulation frequency falls within the 50–120 Hz range shown to be the most efficient for strength training (Hainaut and Duchateau 1992). NMES was produced using selfadhesive bipolar electrodes (4 electrodes for each leg, model MyoTrode, 5×5cm; GLOBUS Italia SRL).. Maximum intensity was achieved by encouraging the patient to bear with the maximum painless level of stimulation, thus reaching a tolerable and effective muscle contraction.

2 negative electrodes were placed close to the proximal insertion of the vastus lateral (VL) and the vastus medialis (VM) muscles, over the femoral triangle of each leg, 1–3cm below the inguinal ligament. Pairs of positive electrodes were placed as close as possible to the motor points of the VL and VM muscles. The motor points were determined by moving a probe over the skin surface to find the lowest threshold for stimulation (Gueldich, Zarrouk et al. 2017). Electrodes were not removed or replaced during the session. All patients were asked to continue their lifestyle as usual. The training procedures were realized by the trained study assistants and supervised by the medical staff.

**Study outcomes**

**Strength measurement**

Participants were seated on an isometric dynamometer (Good Strength, Metitur, Finland) and stabilized with safety belts strapped across the chest, thighs, and hips, to avoid lateral, vertical, or frontal displacements. Participants were seated with a 90° knee fexion angle from full extension with a cuf attached to a strain gauge of the dynamometer. This cuf was adjusted 2 cm above the lateral malleolus using a noncompliant Velcro strap for recording of quadriceps force. All measurements were taken from the participant’s dominant leg.

**Assessments of postural balance**

Participant’s standing postural balance was assessed using a static stabilometric platform (PostureWin©, TechnoConcept®, Cereste, France; 14 Hz frequency, 12-bits A/D conversion) which recorded the displacements of the center of pressure (COP). Participants were instructed to stand erect, as motionless as possible, on a normal comfortable posture, with eyes open looking straight ahead at a cross marked at approximately eye level 3m away and barefoot with feet shoulder width apart on the platform with the arms by their sides and head right. Each participant was requested to keep a quiet stance during 25.6 s following the French Posturology Association norms. To evaluate postural balance of our participants, three COP sways parameters were analyzed in this study: The COP area, the COP lengths corresponding to the sum of COP displacement in the medio-lateral (COPx) and in antero-posterior (COPy)

**Assessments of functional capacity**

**Time Up and Go Test (TUGT)**

Functional mobility was assessed using the Timed Up and Go test (TUGT). The participants were timed as they rose from a 45cm-high straight-backed chair, walked 3m, turned, and returned to their original sitting position (Podsiadlo and Richardson 1991) . The time (s) to accomplish the TUGT was calculated for each participant

**Sit To Stand (STS30)**

Lower-body strength and endurance were determined using the 30-second Sit To Stand test (STS30). Participants were asked to sit in a standard height chair with their arms crossed over the chest, then stand fully and sit down again as many times as possible within 30 seconds (Rikli and Jones 2013)

**Six‐minute walk test (6-MWT)**

The Six-minute walk test (6-MWT) was performed following the recommendations of the American Thoracic Society (Brooks, Solway et al. 2003). During the test, participants were instructed to walk as fast as possible during 6 min on a flat of 30-m long track. They were allowed to stop and to have a rest during the test, but were instructed to resume walking as soon as they felt able to do so. When the test was completed, the total distance travelled was registered.

**Statistical analyses**

The sample size calculation was based on a previous investigation documenting NMES training effects in HD patients compared to control group (Dobsak, Homolka et al. 2012). Assuming an effect size of 0.93, α = 0.05, and β = 0.8, the minimum number of participants required to establish a signifcant difference in maximal voluntary force between before and after NMES intevention and between the two groups using two-way repeated-measures ANOVA, was calculated at 10 per group (G*power, version 3.1.9.4)

Statistical analyses were performed using Statistica for Windows software (version 12.0). The normality of every dependent variable and homogeneity of distribution variances (equal variance) was confirmed using Shapiro-wilk test and the Levene test, respectively. Participant characteristics were compared using independent t-tests. Two-way ANOVA (group x training) was used was used to analyze data. To assess the ANOVA practical significance, partial etasquared (ηp2) was calculated. When a significant difference was found, multiple-comparison analysis was performed with the Bonferroni post hoc test. Results are reported as the mean ± SD and statistical significance was set at P < 0.05.

References

Bowman, B. R. and L. L. Baker (1985). "Effects of waveform parameters on comfort during transcutaneous neuromuscular electrical stimulation." Ann Biomed Eng **13**(1): 59-74.

Brooks, D., S. Solway and W. J. Gibbons (2003). "ATS statement on six-minute walk test." Am J Respir Crit Care Med **167**(9): 1287.

Charlson, M. E., P. Pompei, K. L. Ales and C. R. MacKenzie (1987). "A new method of classifying prognostic comorbidity in longitudinal studies: development and validation." J Chronic Dis **40**(5): 373-383.

Dobsak, P., P. Homolka, J. Svojanovsky, A. Reichertova, M. Soucek, M. Novakova, L. Dusek, J. Vasku, J. C. Eicher and J. Siegelova (2012). "Intra-dialytic electrostimulation of leg extensors may improve exercise tolerance and quality of life in hemodialyzed patients." Artif Organs **36**(1): 71-78.

Gueldich, H., N. Zarrouk, H. Chtourou, F. Zghal, S. Sahli and H. Rebai (2017). "Electrostimulation Training Effects on diurnal Fluctuations of Neuromuscular Performance." Int J Sports Med **38**(1): 41-47.

Hainaut, K. and J. Duchateau (1992). "Neuromuscular electrical stimulation and voluntary exercise." Sports Med **14**(2): 100-113.

Maffiuletti, N. A., G. Cometti, I. G. Amiridis, A. Martin, M. Pousson and J. C. Chatard (2000). "The effects of electromyostimulation training and basketball practice on muscle strength and jumping ability." Int J Sports Med **21**(6): 437-443.

Podsiadlo, D. and S. Richardson (1991). "The timed "Up & Go": a test of basic functional mobility for frail elderly persons." J Am Geriatr Soc **39**(2): 142-148.

Rikli, R. E. and C. J. Jones (2013). "Development and validation of criterion-referenced clinically relevant fitness standards for maintaining physical independence in later years." Gerontologist **53**(2): 255-267.
